# Astrocytic contributions to the pathogenesis of chronic traumatic encephalopathy: a scoping review

**DOI:** 10.1186/s41016-026-00434-w

**Published:** 2026-05-21

**Authors:** Alex Kubiak, Kameron Hahn, Nicholas Powers, Ethan Arroyo, Carson Vandello, Joseph Martins, Ling He

**Affiliations:** Kansas City University School of Medicine, Joplin, MO USA

**Keywords:** Chronic traumatic encephalopathy, CTE, Astrocyte, Tauopathy

## Abstract

**Background:**

Chronic traumatic encephalopathy (CTE) is classically defined as a neuroglial tauopathy characterized by perivascular accumulation of hyperphosphorylated tau within neurons and astrocytes. Defining lesions localize to the depths of cortical sulci, implicating vascular and glial elements in early pathogenesis. This systematic scoping review evaluates the extent to which astrocytic dysfunction contributes to CTE pathogenesis, specifically whether astrocytic alterations precede or potentiate neuronal tauopathy.

**Methods:**

A structured search of PubMed, Embase, Web of Science, and Scopus was conducted from inception through December 2025 in accordance with PRISMA 2020 guidelines. Due to substantial heterogeneity in study design, including postmortem analyses, experimental models, and biomarker studies, a qualitative synthesis was performed.

**Results:**

Forty studies met inclusion criteria, converging on four domains: (1) interface-specific astrogliosis at perivascular sulcal depths; (2) disruption of aquaporin-4 polarization with uncertain translational relevance in humans; (3) astrocytic degeneration and impaired glutamate homeostasis; and (4) sustained neuroinflammation mediated through astrocyte–microglia signaling. Circulating GFAP emerged as a sensitive but non-specific biomarker of neuroglial injury.

**Conclusion:**

CTE is best conceptualized as a neuroglial tauopathy in which astrocytes function as integral contributors to disease progression, likely in coordination with microglia. While astrocytic dysfunction may play an early role, its mechanistic specificity relative to other tauopathies remains incompletely defined.

## Background

Chronic traumatic encephalopathy (CTE) is a progressive neurodegenerative disorder associated with repetitive head impacts through concussive or subconcussive brain injury. It is most commonly encountered in contact sport athletes, military personnel, and others exposed to recurrent mechanical trauma to the brain. Clinically, CTE is characterized by a heterogeneous constellation of cognitive, behavioral, psychiatric, and motor symptoms. Most commonly, this condition is associated with the gradual degeneration of memory and executive function, mood dysregulation with an increase in impulsivity, and depression-like symptomology. Despite growing public awareness and scientific interest, CTE remains a clinicopathological diagnosis with a definitive confirmation currently limited to postmortem neuropathological examination. This diagnostic constraint has substantially impeded efforts to elucidate disease mechanisms through the identification of biomarkers, which could aid in the development of targeted disease-modifying therapies.

Pathologically, CTE is defined by the perivascular accumulation of hyperphosphorylated tau (p-tau) in neurons and astrocytes [1]. While tau pathology has historically been implicated as the prime conceptual framework of CTE pathogenesis, emerging evidence suggests that CTE may not solely be a neuronal tauopathy but rather a complex neuroglial disorder in which astrocytes play a more active role. Astrocytes, the most abundant glial cell type in the central nervous system, are essential for maintaining neuronal homeostasis and integrity, most notably through the regulation of synaptic transmission and neuroinflammatory signaling. Following a traumatic brain injury (TBI), astrocytes undergo dynamic phenotypic changes, which have been referred to as reactive astrogliosis [2]. This process may exert both neuroprotective and neurotoxic effects depending on the severity of the injury and chronicity.

Repetitive brain trauma has been identified as being the primary risk factor for CTE, as it induces sustained astrocytic activation that extends well beyond the acute injury phase. Experimental and human postmortem studies have demonstrated persistent astrocytic hypertrophy through the upregulation of glial fibrillary acidic protein (GFAP). Additionally, altered calcium signaling and dysregulated glutamate clearance may also be contributing factors. These alterations may contribute to excitotoxic injury and chronic neuroinflammation, which are both recognized as key drivers of neurodegeneration. Only recently has it been concluded that astrocytes are known to accumulate pathological tau in CTE [3]. This raises critical questions regarding their role in tau propagation, which can be attributed to clearance failure, particularly at sulcal depths where mechanical strain is greatest.

The specific contributions of astrocytes to CTE pathophysiology remain incompletely defined, and existing studies are heterogeneous in methodology and outcome measures. Much of the literature has emphasized neuronal pathology, with astrocytic changes often relegated to secondary findings. As a result, there is a critical need to systematically synthesize the available evidence examining astrocytic involvement in CTE. This review aims to provide a comprehensive and critical evaluation of the current literature investigating the relationship between astrocytes and CTE. By integrating findings from human neuropathological studies and emerging translational research, this review seeks to clarify the mechanistic roles of astrocytes in CTE development and progression. A more refined understanding of astrocyte-mediated processes in CTE may not only advance the conceptualization of CTE as a neuroglial disorder but also identify novel biomarkers and therapeutic targets relevant to individuals exposed to repetitive head trauma. To address these gaps, this systematic scoping review aims to evaluate the following question: What is the role of astrocytes in the pathogenesis of chronic traumatic encephalopathy, and to what extent do astrocytic mechanisms contribute to disease initiation, propagation, and clinical manifestation relative to neuronal pathology? Given the heterogeneity of available evidence, this review prioritizes mechanistic synthesis over quantitative aggregation.

## Methods

This study was conducted as a review of literature examining the role of astrocytes in the pathophysiology of CTE. The review was designed and reported in accordance with the Preferred Reporting Items for Systematic Reviews and Meta-Analyses (PRISMA) guidelines. Given the predominance of postmortem human neuropathological studies and translational experimental investigations in this field, a qualitative synthesis approach was employed rather than a formal meta-analysis. A quantitative meta-analysis was not performed due to variability in outcome measures, lack of standardized astrocyte-specific endpoints, and heterogeneity in study populations. Future meta-analytic approaches may be feasible for specific outcomes, including circulating GFAP levels, astrocyte density metrics, or region-specific astrocytic pathology, pending standardization across studies. A thorough literature search was performed across multiple electronic databases, including PubMed/MEDLINE, Embase, Web of Science, and Scopus, from database inception through the final search date. The search strategy incorporated both controlled vocabulary terms and free text keywords related to CTE and astrocytic pathology. Our search terms included combinations of “chronic traumatic encephalopathy,” “CTE,” “astrocytes,” “astrogliosis,” “glial pathology,” “glial fibrillary acidic protein,” “GFAP,” “tau,” and “neuroinflammation,” with Boolean operators applied to refine results. To ensure completeness, reference lists of included articles and relevant review papers were manually screened to identify additional eligible studies.

**Fig. 1:**
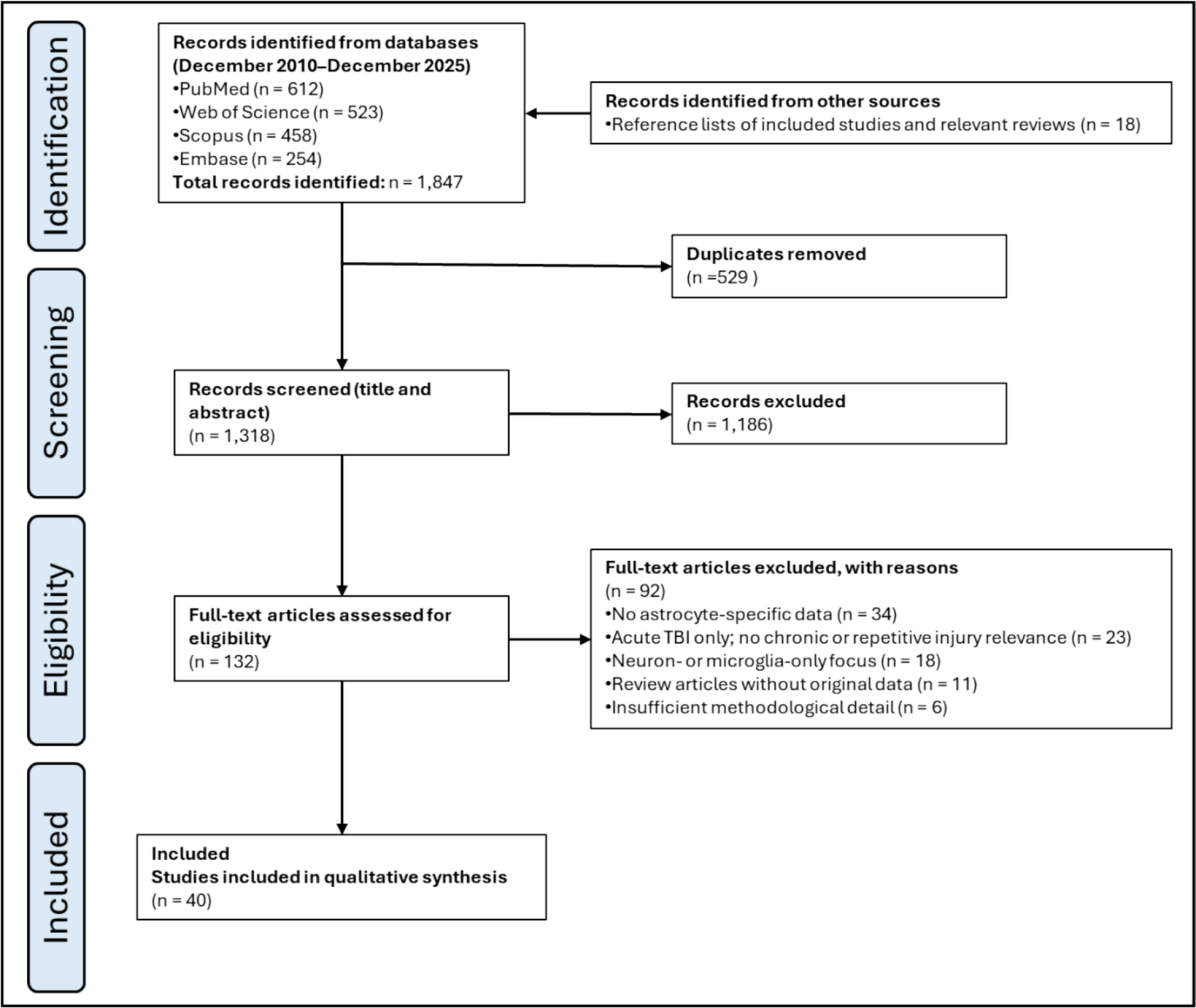
PRISMA flow diagram illustrating study selection

A systematic literature search yielded 1847 records across all databases. After removal of 529 duplicates, 1318 unique records remained for title and abstract screening. Of these, 1186 records were excluded for failing to meet inclusion criteria. The remaining 132 articles underwent a full-text review. Following full-text assessment, 92 studies were excluded for reasons including lack of astrocytic outcome measures, insufficient mechanistic data, or failure to distinguish CTE from other tauopathies. Ultimately, 40 studies met all inclusion criteria and were included in the final qualitative synthesis. The study selection process is summarized in a PRISMA flow diagram (Fig. 1). Although the final number of included studies was limited, this outcome is consistent with the highly specific inclusion criteria and the underrepresentation of astrocyte-centered investigations within the broader CTE literature. All identified records were imported into a reference management system. Any discrepancies in study selection were resolved through discussion and consensus to ensure consistent application of the inclusion and exclusion criteria. Study identification, screening, and eligibility assessment were performed exclusively through manual review by the investigators. No AI-assisted tools, automated screening platforms, or algorithmic prioritization methods were employed. All abstracts and full-text articles were independently read and evaluated by human reviewers to ensure transparent and unbiased study selection.

Specifically, studies were considered eligible for inclusion if they investigated astrocytes or astrocyte-related pathological processes in the context of CTE or repetitive TBI with relevance to CTE neuropathology. Eligible study designs included human postmortem analyses, animal models, and in vitro translational studies that explicitly addressed astrocytic mechanisms. They were determined to be relevant if they examined at least one mechanistic domain of astrocytic dysfunction. Our most relevant topics included glymphatic clearance, EAAT2-mediated excitotoxicity, metabolic or oxidative stress, biomechanical patterning, tau pathology, or inflammatory transformation. Studies were excluded if they focused exclusively on non-glial aspects of CTE. Only peer-reviewed articles published in English were included, as we were not certain if our translations were accurate.

After a title and abstract review, studies were explicitly excluded if they focused exclusively on neuronal pathology without astrocytic data, addressed acute TBI without relevance to chronic exposure, or examined unrelated tauopathies without reference to CTE. We found that there was a limited number of eligible studies which reflects the conceptual novelty and narrow mechanistic focus of this review rather than deficiencies in the existing literature. While numerous studies address traumatic brain injury and tau pathology broadly, comparatively few directly interrogate astrocyte-specific mechanisms in the context of chronic traumatic encephalopathy, resulting in a smaller but thematically coherent evidence base. After a full-text assessment, articles were excluded only after a comprehensive evaluation against predefined inclusion and exclusion criteria. The most common reasons for exclusion included the absence of astrocyte-specific outcome measures or mechanistic analyses; failure to differentiate chronic traumatic encephalopathy from other tauopathies or neurodegenerative disorders; descriptive pathology without spatial, molecular, or functional characterization of astrocytic involvement; insufficient methodological detail to support mechanistic inference; and non-primary research designs, including narrative reviews, editorials, or opinion pieces. To ensure transparency and accountability, all excluded full-text studies were logged with a prespecified primary reason for exclusion. Exclusion decisions were made through manual, article-level assessment, and studies were excluded only when they failed to meet one or more inclusion criteria despite initial relevance. This approach minimized erroneous exclusion while maintaining the methodological thematic specificity.

Data was extracted using a standardized framework to ensure uniformity across studies. Extracted information included study design and experimental model. We took significant inspiration from Maroon et al. [4] as we felt that they had done a remarkable job examining CTE from various clinical sources [4]. We wanted to replicate that by using sample characteristics, such as an astrocytic marker assessment. This includes glial fibrillary acidic protein and tau isoforms, the anatomical and spatial distribution of astrocytic pathology with reported associations with tau deposition, and neuroinflammation. Key mechanistic findings relevant to astrocyte function and dysfunction in CTE were also recorded. Extracted information included study design, brain region analyzed, astrocyte-related outcomes, mechanistic domain, and key findings. The most relevant excluded information included the mechanism of injury involving the TBI or CTE, the various ages of the subjects within the referenced studies, and any prior variables relating to the health of the subjects indicated within each sample. It was decided that such information was not pertinent or supportive to the conclusion of the review.

Methodological quality was assessed qualitatively due to the lack of validated risk of bias tools specific to CTE neuropathology. Studies were evaluated based on the clarity of CTE diagnostic criteria. From our research, this is most adequately reported through tissue sampling and regional analysis, and the use of validated immunohistochemical markers. Given the substantial heterogeneity across study designs, a quantitative synthesis was not performed. Instead, findings were synthesized narratively and organized into eight thematic domains, which include foundational analysis using histopathological, qualitative, and molecular evidence; mechanobiology and glymphatic dysfunction; glutamate homeostasis and synaptic failure; neuroinflammation and toxic astrocyte phenotypes; comparative pathology (CTE vs. ARTAG vs. AD); translational biomarkers and clinical relevance; stage-specific and regional astrocytic profiling; and astrocyte-centered pathogenesis in CTE.

Convergent and divergent findings were critically evaluated to identify emerging mechanistic patterns and persistent gaps in the current literature. The methodological rigor of the included studies was systematically evaluated using criteria tailored to neuropathological and preclinical research. Key quality indicators included sample size adequacy, inclusion of control groups, implementation of blinding procedures, replication of findings, and comprehensive reporting of outcomes. Each study was subsequently categorized as high, moderate, or low quality. This is what informed the relative weighing of evidence during data synthesis. Such a structured assessment enabled a domain-specific analysis of astrocytic dysfunction in CTE while enhancing the transparency and reliability of the review’s conclusions.

## Results

### Study selection

The database search identified 1847 records. After removal of duplicates, 1318 records were screened, and 132 full-text articles were assessed for eligibility. Forty studies met the inclusion criteria. A PRISMA 2020 flow diagram summarizing the study selection process is provided (Fig. 1).

### Risk of bias

The overall risk of bias was assessed using the Newcastle–Ottawa Scale (NOS) for human observational studies and the SYRCLE risk of bias tool for animal studies. Across human postmortem investigations, selection bias remained a prominent concern due to reliance on brain bank donations and retrospective exposure ascertainment; however, studies that incorporated age-matched control groups and explicitly accounted for comorbid neurodegenerative pathologies demonstrated lower risk in the comparability domain of the NOS. Particular attention was paid to whether neuropathological assessments were performed blinded to subjects’ athletic or exposure histories, as lack of blinding represents a recognized source of ascertainment bias in CTE research. Animal studies were generally at low risk of selection bias but showed variable risk related to allocation concealment and blinding of outcome assessment under SYRCLE criteria. Risk of bias evaluations were conducted independently by multiple reviewers, with discrepancies resolved by consensus, strengthening the internal validity of the assessment while acknowledging that methodological heterogeneity and limited sample sizes continue to constrain causal inference and generalizability.

To promote methodological accountability, each included study underwent a qualitative assessment of risk of transparency bias at the individual study level. Transparency bias was defined as insufficient disclosure of study design, methodology, data reporting, or interpretive rationale that could limit reproducibility or critical appraisal. Using a predefined framework, studies were evaluated across multiple domains, such as the methodological clarity and completeness, risk of selective outcome reporting, and adequacy of conflict-of-interest and funding disclosures. Each domain was rated as low, moderate, or high risk, and an overall transparency risk judgment was assigned accordingly. Transparency risk did not serve as an exclusion criterion; rather, these assessments were used to contextualize the strength and interpretability of mechanistic conclusions during qualitative synthesis.

### Domain A: foundational analysis using histopathological, qualitative, and molecular evidence

Osterman et al. [5] substantially refined the pathological framework of CTE by identifying perivascular glial reactivity as an essential constituent of the phosphorylated tau (p-tau) lesion [5]. Their findings demonstrated that astrocytic processes predominate at the earliest and most anatomically restricted sites of tau accumulation. More specifically, they were frequently discovered at the depths of cortical sulci, where biomechanical stress is greatest during repetitive head injury. This observation may contribute to reframing CTE as a disorder rooted in astrocytic dysfunction and failed neurovascular support. Complementing this conceptual shift, Hsu et al. [6] provided mechanistic evidence of astrocytic degeneration through the description of clasmatodendrosis, which is most characterized by cytoskeletal fragmentation and impaired ionic homeostasis [6]. Notably, astrocytic degeneration was discovered to precede overt neuronal loss. This supports a model in which neuronal vulnerability in CTE arises secondarily from compromised glial integrity rather than a dysfunction of the intrinsic neuronal pathology.

With recent extensions of histopathological observations into the molecular domain, Suter et al. [7] employed spatial transcriptomics to define disease-specific astrocyte signatures in CTE tissue. These astrocytes exhibited coordinated dysregulation of glutamate transport, inflammatory signaling cascades, and cytoskeletal maintenance pathways [7]. This may explicitly implicate functional deficits in synaptic regulation and neuroinflammatory control. Importantly, the transcriptional profiles of CTE-associated astrocytes were distinct from those observed in Alzheimer’s disease (AD) and aging-related tau astrogliopathy (ARTAG). This may provide a clear molecular validation that astrocytic pathology in CTE represents a unique glial phenotype rather than a nonspecific response to tau accumulation. These findings converge on a unifying framework in which astrocytic failure could potentially serve as a central driver of CTE pathogenesis (Table 1).

**Table 1:** Foundational histopathological, qualitative, and molecular evidence

Study	Model/cohort	Astrocytic feature assessed	Key findings	Spatial pattern	Interpretation for CTE pathogenesis
(Osterman et al. [5])	Human postmortem CTE	Reactive astrogliosis, oxidative stress markers	Astrocytes integral to perivascular p-tau lesions; NQO1⁺ and L-ferritin⁺ astrocytes indicate oxidative stress	Perivascular, sulcal depths	Astrocytes are structural components of the pathognomonic CTE lesion
(Hsu et al. [6])	Human postmortem CTE vs. AD/FTD	Astrocytic degeneration (clasmatodendrosis)	Clasmatodendrosis present in 71% of CTE cases; precedes neuronal loss	Diffuse white matter > gray matter	Astrocytic structural failure precedes neuronal vulnerability
(McKee et al. [8])	Young athletes with early CTE	Interface astrogliosis	Subpial and gray–white junction astrogliosis present early	Interface regions	Astrocytic changes occur early, even with limited exposure
(Mez et al. [9])	Large CTE case series	Astrocytic tau pathology	Astrocytic p-tau increases with CTE stage	Subpial and perivascular	Astrocytic tau is stage-dependent and diagnostic

### Domain B: mechanobiology and glymphatic dysfunction

Astrocytic pathology in CTE is most coherently contextualized through disruption of the glymphatic system. This system has been identified as an astrocyte-dependent perivascular clearance pathway critical for metabolic waste removal. Iliff et al. [10] first characterized the glymphatic system as reliant on polarized expression of aquaporin-4 (AQP4) channels, which enabled cerebrospinal and interstitial fluid exchange [10]. This has been noted as the most essential pathway for the clearance of interstitial solutes, including tau; evidence supporting a central role for glymphatic dysfunction in human CTE remains limited. This framework may provide a mechanistic explanation for the accumulation of phosphorylated tau observed in CTE lesions. Subsequent experimental work demonstrated that a TBI directly impairs glymphatic function. This is most consistently observed in Iliff et al. [11], which showed that mechanical injury disrupts AQP4 polarization and leads to reduced glymphatic flux through the acceleration of tau aggregation in vivo [11]. Complementary findings by Plog et al. [12] further established that the loss of AQP4 polarity following a TBI markedly diminished metabolic waste drainage [12]. This perfectly cultivates a permissive microenvironment for progressive proteinopathy. Together, these studies position the absence of astrocytic AQP4 as a proximal event linking mechanical injury to impaired clearance and tau accumulation. Since these studies were conducted in mouse models, further research is needed to establish glymphatic alterations as a central mechanism to astrocyte dysfunction in humans.

Astrocytic pathology in CTE appears to be spatially patterned by biomechanical forces rather than random distribution. A neuropathological analysis by Shively et al. [13] identified pronounced astrogliosis at gray and white matter junctions [13]. Interestingly, these anomalies had been previously predicted by computational models that simulated maximal shear strain during a rapid acceleration–deceleration injury. Extending this paradigm, Daneshvar et al. [14] correlated head impact kinematics with the spatial distribution of astrocytic p-tau pathology, reinforcing the concept that astrocytic injury reflects a region-specific mechanical vulnerability [14]. Glymphatic flow has been shown to be preferentially concentrated along perivascular spaces susceptible to mechanical distortion [15, 16], while age and injury-related AQP4 depolarization further exacerbates clearance deficits [17]. These findings integrate astrocytic degeneration with glymphatic failure into a unified pathogenic axis. With repetitive head trauma, there may very well be an inducible and regionally patterned astrocytic dysfunction that precedes and facilitates tau accumulation in CTE (Table 2).

**Table 2:** Mechanobiology and glymphatic dysfunction

Study	Model	Astrocytic mechanism	Key findings	Functional consequence
(Iliff et al. [10])	Rodent	AQP4-mediated glymphatic flow	Polarized AQP4 required for CSF–ISF exchange and tau clearance	Establishes astrocyte-dependent clearance system
(Iliff et al. [11])	Rodent TBI	AQP4 depolarization	TBI disrupts AQP4 polarity and reduces glymphatic flux	Impaired tau clearance post-injury
(Shively et al. [13])	Human postmortem + modeling	Mechanically patterned astrogliosis	Astrogliosis localizes to regions of maximal shear strain	Astrocytic injury follows biomechanical forces

### Domain C: glutamate homeostasis and synaptic failure

Astrocytes are critical regulators of synaptic glutamate homeostasis mediated through the excitatory amino acid transporter 2 (EAAT2). Loss or dysfunction of EAAT2 disrupts glutamate clearance, which is linked to excitotoxic neuronal injury [18]. In CTE, astrocytic EAAT2 downregulation provides a correlation between repetitive head trauma and persistent synaptic dysfunction. These findings may indicate that neuronal pathology may be secondary to impaired glial support. Beyond glutamate excitotoxicity, astrocytes interact with other glial populations to maintain neural network stability. Chancellor et al. [19] demonstrated coordinated astrocyte and oligodendrocyte alterations in CTE, emphasizing that disease pathogenesis extends beyond neurons to the interconnected glial network [19]. These findings signify the possibility of considering CTE as a glial-centric disorder in which disruptions between the astrocyte and oligodendrocyte networks contribute to neurodegenerative progression. Astrocytic metabolic dysfunction further amplifies disease vulnerability. Shin et al. [20] identified endothelial nitric oxide synthase (eNOS) uncoupling as a contributor to oxidative stress and p-tau accumulation within astrocytes [20]. It is possible that this is a direct implication of the disrupted astrocyte and vascular metabolic coupling, which can act as a driver of CTE pathology. These observations align with broader evidence that astrocytes serve as metabolic buffers that have been noted to regulate redox homeostasis and ion balance within neural circuits [21, 22]. Loss of these regulatory capacities may synergize with excitotoxic stress to accelerate synaptic and axonal degeneration (Table 3).

**Table 3:** Glutamate homeostasis and synaptic failure

Study	Model	Astrocytic function	Key findings	Pathophysiologic implication
(Chancellor et al. [19])	Human postmortem snRNA-seq	Glial network regulation	Coordinated astrocyte–oligodendrocyte inflammatory signaling	Network-level glial dysfunction
(Cho et al. [23])	Human transcriptomics	Synaptic gene regulation	Downregulation of synaptic and memory-related genes; CAMs upregulated in CTE	Astrocyte-linked synaptic destabilization

### Domain D: neuroinflammation and toxic astrocyte phenotypes

The inflammatory transformation of astrocytes represents a critical pathogenic inflection point in CTE. Historically, astrocytes were divided into A1 neurotoxic and A2 neuroprotective phenotypes. Liddelow et al. [24] defined the A1 neurotoxic astrocyte phenotype as inducible by microglia-derived proinflammatory cytokines, which include interleukin-1α, TNF-α, and complement component C1q [24]. A1 astrocytes were characterized by the loss of synaptotrophic support and the gain of neurotoxic functions that actively promote neuronal death. More recently, the A1 and A2 dichotomy has transitioned to a spectrum of astrocyte inflammatory activity. This framework provides an interpretation of how immunological function through astrocytic activation contributes to localized tau pathology and synaptic dysfunction in CTE. Supporting this concept, Cherry et al. [25] demonstrated that microglial activation not only precedes but also spatially predicts astrocytic tau deposition in postmortem CTE tissue [25]. This may suggest a coordinated association between microglia and astrocyte signaling. These findings may also imply that the early innate immune response to repetitive head trauma can propagate astrocytic neurotoxicity in a region-specific manner. In the case of CTE, it is particularly localized at the depths of cortical sulci. Emerging molecular and transcriptomic studies further elucidate the role of inflammatory astrocytes in CTE. Ruchika et al. [26] synthesized data to show that chronic glial activation persists well beyond the initial traumatic event [26]. This discovery signifies the possible existence of a self-perpetuating cycle in which astrocytes act as both effectors and amplifiers of neuroinflammation. After chronic exposure to head trauma, there is a disruption to normal cytokine signaling and reactive oxygen species clearance and complement pathway activation. These collectively create an environment that favors tau hyperphosphorylation and neuronal vulnerability. In addition, recent evidence indicates that the balance between neurotoxic astrocytic activity is dynamically regulated by the microenvironment. While there has been some speculation as to the significance of the neurotoxic astrocyte phenotype in CTE, we believe that there may be more to these findings that have not yet been effectively consolidated and concluded. According to the literature, with repetitive traumatic events to the CNS, there is an imbalance that propels the microenvironment toward a sustained neurotoxic state [27, 28]. This dynamic may further imply the concept that astrocytes are not passive responders to neuronal injury but active participants in disease propagation. The inflammatory transformation of astrocytes integrates microglial signaling, oxidative stress, and tau pathology into a unified pathogenic axis where astrocytic inflammation may be positioned as a central driver of neurodegeneration in CTE. Still, it should be noted that the data relating to astrocytic neuroinflammation is shared between many neurodegenerative disorders and is not specific to CTE (Table 4).

**Table 4:** Neuroinflammation and toxic astrocyte phenotypes

Study	Model	Astrocytic phenotype	Key findings	Mechanistic insight
(Liddelow et al. [24])	Experimental	Neurotoxic astrocytes	Microglial cytokines induce neurotoxic astrocytes	Defines toxic astrocyte framework relevant to CTE
(Cherry et al. [25])	Human postmortem CTE	Microglia–astrocyte coupling	Microglial activation predicts astrocytic tau	Inflammatory crosstalk drives pathology

### Domain E: comparative pathology (CTE vs. ARTAG vs. AD)

Differentiating CTE from other tauopathies is essential for diagnostic validity and for avoiding pathological conflation. Kovacs et al. [29] defined ARTAG as an age-associated astrocytic tauopathy characterized by subpial and periventricular astrocytic tau deposition that follows anatomical distributions independent of traumatic exposure [29]. Although ARTAG and CTE both involve astrocytic tau pathology, their spatial patterns diverge substantially. Arena et al. [30] provided decisive phenotypic clarification by demonstrating that astrocytic tau in CTE exhibits a highly specific localization to perivascular regions at the depths of cortical sulci, a unique feature that is not observed in AD or ARTAG [30]. This sulcal depth may reinforce the mechanistic link between biomechanical stress and astrocytic vulnerability unique to CTE. These pathological distinctions were formalized by McKee et al. [31] through the National Institute of Neurological Disorders and Stroke (NINDS) consensus diagnostic criteria for CTE [31]. This has established perivascular p-tau aggregates in neurons and astrocytes at specific sulcal depths. By standardizing astrocytic pathology within a reproducible neuropathological framework, the NINDS criteria have elevated astrocytes to one of the primary responders to disease-defining elements of CTE. Collectively, this body of work signifies that astrocytic tau pathology in CTE is not a nonspecific manifestation of aging or neurodegeneration; instead, it is a spatially and phenotypically distinct process critical for diagnostic specificity. These distinctions are fundamental for both accurate postmortem diagnosis and the development of future in vivo biomarkers capable of reliably discriminating CTE from other tauopathies. The interface between CTE and ARTAG remains an area of ongoing uncertainty. Both conditions involve tau deposition and may even occur simultaneously. While spatial patterning may be a suitable discriminator, diagnostic criteria and molecular signatures must be refined to truly differentiate these similar tauopathies clinically (Table 5).

**Table 5:** Comparative pathology (CTE vs. ARTAG vs. AD)

Study	Comparison	Astrocytic tau pattern	Key distinction
(Arena et al. [30])	CTE vs. ARTAG vs. AD	Thorn-shaped astrocytes at sulcal depths	Spatial localization, not tau isoform, distinguishes CTE
(McKee et al. [31])	Consensus criteria	Perivascular astrocytic tau	Astrocytes embedded in diagnostic lesion

### Domain F: translational biomarkers and clinical relevance

Blood-based biomarkers have emerged as a critical translational bridge between the molecular pathology of brain injury and clinically actionable diagnostics. Zetterberg et al. (2016) provided early and influential evidence that circulating proteins such as GFAP and tau rise after head trauma, which provides a link between astroglial injury and measurable peripheral signals [17]. The increasing presence of GFAP reflects acute astroglial disruption and BBB compromise, whereas tau elevations index neuronal and axonal damage. McCunn et al. [32] further refined this framework by emphasizing the GFAP cell-type specificity within cases of high-impact head trauma, supporting its role as a mechanistically grounded biomarker [32]. Together, these studies underscore the translational value of GFAP-centered biomarker panels in improving diagnostic objectivity and potentially informing clinical decisions. Unfortunately, biomarkers like GFAP can be altered by any brain injury, which makes it difficult to find markers specific to CTE. Importantly, biomarkers such as GFAP and neurofilament light chain are not specific to CTE and may be elevated in a wide range of neurological insults, including acute traumatic brain injury.

Beyond acute injury, translational biomarker research is increasingly motivated by the need for antemortem diagnosis of CTE. Alosco et al. [33] framed this challenge as the “holy grail” of the field [33]. The study highlights the gap between post-mortem neuropathologic criteria and the absence of validated in vivo diagnostic tools. While blood biomarkers such as tau and GFAP capture aspects of neurodegeneration, their lack of specificity for CTE further amplifies the necessity of a multimodal approach for integrating fluid biomarkers and clinical phenotyping. Collectively, these studies illustrate the current limitations of translational biomarkers. They are able to provide clinically relevant signals of brain injury; however, they are insufficient for a definite CTE diagnosis. Ongoing refinement of biomarker specificity represents a central translational priority in neurodegenerative disease research (Table 6).

**Table 6:** Translational biomarkers and clinical relevance

Study	Biomarker	Sample	Key finding	Clinical relevance
(Zetterberg & Blennow [17])	GFAP	Blood	GFAP rises after head trauma	Sensitive marker of astroglial injury
(McCunn et al. [32])	GFAP	Blood	GFAP reflects astrocyte-specific injury	Translational astrocytic biomarker

### Domain G: stage-specific and regional astrocytic profiling

Disease progression in CTE is increasingly understood as a stage-dependent process in which astrocytes play an early and active role. Proteomic profiling by Gutierrez-Quiceno et al. [34] identified stage-specific astrocytic molecular signatures in postmortem CTE tissue, demonstrating that astrocytic stress responses and cytoskeletal alterations emerge prior to widespread tau aggregation [34]. These findings may suggest that early astrocytic dysfunction has the potential to shape regional vulnerability and permissive environments for subsequent tau aggregation. Consistent with this framework, McKee et al. [8] demonstrated astrocytic abnormalities in young contact-sport athletes with relatively limited cumulative exposure, indicating that astrocytes are among the earliest cellular responders to repetitive head impact [8]. As CTE advances, astrocytic pathology appears to evolve toward persistent inflammatory and degenerative phenotypes that may contribute to chronic disease maintenance. Stein et al. [35] expanded the pathological spectrum of CTE by identifying glial TDP-43 pathology that strongly implicates astrocytes and other glial populations in proteinopathies beyond tau alone [35]. Experimental evidence from Tagge et al. [36] demonstrated that even a single concussive event can induce long-lasting astrocytic remodeling in animal models [36]. Extending these observations to human disease, He et al. [37] reported persistent upregulation of inflammatory and immune-related gene expression in astrocytes isolated from CTE brains [37]. This reinforces the concept that astrocytes actively sustain chronic neuroinflammation rather than reflecting downstream damage. Collectively, these studies have the potential to contribute in reframing astrocytes as regionally and temporally heterogeneous drivers of CTE pathology (Table 7).

**Table 7:** Stage-specific and regional astrocytic profiling

Study	Model/cohort	Disease stage or exposure	Brain regions examined	Astrocytic features identified	Temporal/regional implication
(Gutierrez-Quiceno et al. [34])	Human postmortem proteomics	CTE stages I–IV	Multiple cortical and subcortical regions	Stage-specific astrocytic stress-response and cytoskeletal protein alterations	Astrocytic molecular changes emerge early and evolve with disease progression
(McKee et al. [8])	Young contact-sport athletes	Early-stage/limited exposure	Cortical sulci, subpial regions, white matter	Interface astrogliosis and early astrocytic tau pathology	Astrocytes are early responders prior to widespread tau burden
(Tagge et al. [36])	Experimental repetitive head impact	Single and repetitive injury	Diffuse cortical and subcortical regions	Persistent astrocytic remodeling after single concussion	Astrocytic alterations can be long-lasting and precede neurodegeneration
(Mez et al. [9])	Large clinicopathological cohort	Stage I–IV CTE	Cortex-wide (standardized sampling)	Increasing astrocytic p-tau with advancing stage	Astrocytic pathology tracks with disease severity

### Domain H: astrocyte-centered pathogenesis in CTE

Several foundational works contextualize CTE pathogenesis without an astrocyte-centered interpretation of the disease. Murray et al. [38] provided a comprehensive overview of CTE pathobiology, integrating tau aggregation, neuroinflammation, and clinical progression; however, astrocytes were treated largely as secondary components rather than as active drivers of disease [38]. This is one of many studies that highlight a critical gap that the present review aims to directly address. Quantitative evidence supporting astrocytic prominence was provided by Arena et al. [30], who compared glial density across AD and CTE brains and demonstrated disproportionately elevated astrocytic responses in CTE [30]. The research suggested disease-specific glial activation patterns rather than nonspecific neurodegenerative gliosis. Expanding the mechanistic scope, Akbari-Gharalari et al. [39] signified the existence of glial-derived exosomes, which are small extracellular vesicles that are released by glial cells that act as potential mediators of tau propagation [39]. This further implicates astrocytes in both local pathology and the intercellular spread of disease-relevant protein aggregation.

The clinical significance of astrocyte-linked pathology is reinforced by large-scale clinicopathological studies. Mez et al. [9] analyzed 202 former American football players and demonstrated strong correlations between neuropathological burden and clinical symptomatology [9]. This provides a robust human framework in which astrocytic pathology may plausibly contribute to neuropsychiatric and cognitive decline. Finally, McKee et al. [40] offered an authoritative consensus on CTE pathology in relation to diagnostic challenges [40]. When viewed collectively, these studies establish astrocytes as key role players in CTE, accompanying neuron and microglial pathology as observed in other tauopathies like AD (Table 8).

**Table 8:** Astrocyte-centered pathogenesis in CTE

Study	Evidence type	Astrocytic mechanism	Key findings	Implication for disease model
(Arena et al. [30])	Comparative neuropathology	Disproportionate astrocytic activation	Astrocytic responses are more prominent in CTE than AD	Supports disease-specific astrocyte involvement
(Mez et al. [9])	Clinicopathological correlation	Astrocytic tau and gliosis	Astrocytic pathology correlates with clinical symptom burden	Astrocytes contribute to clinical expression of disease
(Akbari-Gharalari et al. [39])	Mechanistic synthesis	Glial-derived exosomes	Astrocyte-derived exosomes may mediate tau propagation	Astrocytes participate in intercellular spread of pathology
(McKee [40])	Consensus framework	Integrated neuroglial pathology	CTE defined by combined neuronal and astrocytic lesions	Positions astrocytes as central pathogenic drivers

## Discussion

This review compiles the most current evidence implicating astrocytes as central contributors to the pathogenesis of CTE. A consistent pattern is present in which astrocytic pathology is not merely reactive or secondary to neuronal degeneration but instead may represent a defining and potentially initiating factor of CTE. The reviewed literature demonstrates that astrocytes in CTE exhibit distinctive phenotypes, which include accumulation of p-tau, profound reactive astrogliosis, astrocytic degeneration characterized by clasmatodendrosis, and disruption of astrocyte-dependent clearance. Importantly, these features appear spatially and temporally linked to the hallmark neuropathological lesions present within post-mortem diagnoses of CTE. They distinguish the disorder from other tauopathies, reinforcing the relevance of astrocyte-centered disease models. Importantly, many of the astrocytic and neuroinflammatory mechanisms described in this review are not unique to CTE and are also observed in other tauopathies, including Alzheimer’s disease. This overlap complicates mechanistic attribution and highlights the need to distinguish disease-specific spatial patterning, particularly the perivascular and sulcal distribution characteristic of CTE, from shared molecular pathways.

### Clinical implications

The findings of this review support the reframing of CTE from being a predominantly neuron-centric tauopathy to a disorder that involves both neuroglial and neurovascular unit dysfunction. Countless postmortem studies support the claim of astrocytic pathology in CTE. The different pathologies include reactive astrogliosis, astrocytic tau accumulation, and degenerative phenotypes. Each of these illustrates a defining feature of the CTE lesion that typically occurs in perivascular regions at sulcal depths [31]. This pattern of astrocyte involvement aligns with the biomechanical models of injury and helps to reinforce the concept that astrocytes are often the earliest and most at-risk cellular responders to repetitive head impacts. Clinically, this framework of astrocyte involvement can have significant implications for both diagnosis and treatment. Currently, CTE can only be definitively diagnosed postmortem due to the need to examine brain tissue. Therefore, the main diagnosis goal in clinical care should not be to confirm that a living individual has CTE, but instead identify individuals who are at a higher risk and monitor them over time [8]. A significant number of large studies have found a correlation between the amount of brain damage found postmortem and the neuropsychiatric and cognitive impairment an individual had while living. This illustrates the importance of finding biological warning signs before permanent brain damage occurs. Therefore, using astrocyte aggregation as a biological marker to screen for CTE can be an effective technique due to the fact that they are one of the primary responders to head trauma [9].

Various studies conclude that markers released by astrocytes can play a major role in the diagnosis of CTE. One of the main markers discussed is GFAP, one that indicates astrocyte involvement in head trauma [17]. Although GFAP isn’t linked to a specific disease, its linkage to astrocytes makes it a great biomarker for monitoring neuroglial injury and its relation to CTE. Prior studies emphasize that CTE cannot be identified through one single test and, therefore, physicians combine injury history, symptoms, and blood markers [33]. Within these frameworks, astrocytic markers can provide an additional approach in the diagnosis and further understanding of CTE. Furthermore, the correlation between astrocytes and CTE has various clinical correlations. Research suggests that astrocytic dysfunction often precedes or parallels neuronal degeneration [24]. This research points to the fact that using astrocytes can be effective in the early disease course of CTE. Additionally, it may indicate that immune cells in the brain promote astrocytes’ transition into aggressive states. These areas of immune overactivation often parallel astrocyte-related damage seen in patients with CTE [25].

Aside from an improved understanding of the role of astrocytes in the pathogenesis of CTE, this review also identifies additional processes linked to astrocytes that can guide future antemortem diagnostic tools. In regards to postmortem settings, diagnostic precision can be improved by emphasizing astrocytes as integral to the pathogenesis of CTE lesions instead of a supporting role. The localization of p-tau accumulation, neurodegenerative astrocytic phenotypes, and reactive astrogliosis to regions of the brain could simultaneously contextualize and reinform that consensus criteria for CTE diagnosis. Through the integration of astrocytic biomarkers, CTE can be better distinguished from other tauopathies, including Alzheimer’s disease. A further understanding of astrocytes in CTE may also aid in identifying gene signatures that could refine staging and reduce diagnostic ambiguity with comorbid neuropathology.

Collectively, by putting together postmortem pathology with biomarker research, this review narrows the conceptual gap between the definitive diagnosis after death and antemortem detection. It establishes astrocytes as a critical link between mechanical brain injury and peripheral signals, thereby providing a coherent foundation for improving diagnostic accuracy and specificity in CTE. This mechanism provides possible targets for advancements in imaging or biomarker panels aimed at detecting CTE pathology before irreversible damage occurs. This would allow the diagnostic process to move from postmortem confirmation of end stage disease to proactive identification of CTE in individuals based on early neuroglial dysfunction.

### Limitations of evidence

Despite growing research, several limitations make interpretation difficult. A majority of the available data related to CTE stems from postmortem brain tissue, which limits the ability to infer and determine the causation of CTE. These deductions can only be conclusively made when the methods of diagnosis apply to living patients. Without diagnostic capabilities, biological markers detected in postmortem tissue can only serve as correlates. In addition, sample sizes are often small, and cohorts frequently lack diversity in terms of sex, race, and exposure history. This may cause limitations to the findings by reducing the scope and applicability. Additionally, comorbid neuropathologies are difficult to fully disentangle. Several aforementioned inflammatory markers, such as TNF-α and IL-α, are present in other.

neuroinflammatory pathologies. Patients with CTE often simultaneously suffer from Alzheimer’s disease [35]. Difficulty differentiating the pathological mechanisms and biological markers between neuroinflammatory diseases limits the ability to conclusively attribute inflammatory markers solely to CTE in cases involving multiple neuropathies. Heterogeneity of study populations and protocols restricts the ability to draw definitive conclusions for antemortem diagnosis. As mentioned, comorbid neuropathies could present with similar biological markers, including changes with astrocytes. Many of the studies did not address the inclusion or exclusion of patients with a comorbid diagnosis. Additionally, variability in neuropathological diagnostic criteria and inter-observer interpretation introduces further uncertainty in study comparisons and may influence reported astrocytic involvement. The ability to diagnose CTE in a living patient could be impacted by other diseases; thus, comorbid neuropathologies must be investigated further before astrocytes can definitively be used for diagnosis.

### Limitations of the review process

Throughout our research process, only studies published in English were included, which may introduce language bias and limit global generalizability. Future reviews incorporating multilingual databases may capture emerging data from regions with expanding CTE research and improve the comprehensiveness of evidence synthesis. This reduced the available data that could be used to draw a more definitive correlation of astrocytes with CTE. In subsequent future research, we would like to include studies in other languages to avoid language bias, lack of generalization, and the possible exclusion of unique findings. Specifically, we are interested in a recent emergence of premortem animal-model studies from India and China that replicated CTE symptomatology and studied various correlations.

### Future directions

One of the major limitations of existing literature on CTE is that it consistently relies on postmortem analysis, which makes it difficult to interpret the order in which detrimental changes happen to the brain. Therefore, future studies should prioritize tracking individuals on a long-term basis to fully understand the complex pathogenesis of CTE. The research should integrate exposure history, clinical trajectories, and serial biomarker assessment in order to completely understand the astrocytic dysfunction that may contribute to CTE. Early research suggests that changes in astrocytes appear early and continue to worsen and evolve as the disease progresses [8]. Therefore, expanding the research across larger and more diverse cohorts will be essential for establishing a concrete timeline of CTE development and identifying the most appropriate windows for intervention. As stated previously, current research methods suggest astrocytes behave differently in CTE than they do when compared to Alzheimer’s disease or normal aging [19]. Future studies should focus on further distinguishing between these astrocyte-specific molecular signatures in order to improve diagnosis of CTE. Establishing reproducible astrocytic disease signatures can lead to an improvement in diagnostic specificity, as well as understanding the biological steps causing CTE. The issue in differentiating CTE from other tauopathies remains a difficult challenge and it is one that must be fully explored in future research. Specifically, emerging therapeutic strategies targeting astrocytic function warrant further investigation. These include modulation of aquaporin-4 (AQP4) to restore perivascular clearance, upregulation of EAAT2 to improve glutamate homeostasis, and interventions aimed at attenuating maladaptive astrocyte-mediated inflammatory responses. While these approaches remain largely preclinical, they represent promising avenues for disease modification.

Overall, translational, neuropathological, and molecular evidence all point to the idea of using astrocytes as therapeutic targets in CTE. Most therapeutic approaches focus on keeping astrocytes functioning normally. This would be best observed through their ability to clear excess chemical signals, maintain waste removal, and avoid harmful inflammatory states [25]. Each of these focuses offers new possible treatment options for CTE. In order to progress the therapeutic research of CTE, it will require coordinated research with the development of reliable biological tests and the long-term monitoring of patients.

## Conclusion

CTE has long been interpreted through a neuron-centric framework with tau aggregation positioned as both the defining lesion and the primary driver of disease. The evidence gathered in this review challenges that paradigm. Across neuropathological and biomechanical studies, a convergent pattern emerges in which astrocytes may not be the passive responders to neuronal injury as hypothesized. Instead, they may contribute to CTE as early and active participants in the disease process. The perivascular distribution of CTE lesions, the vulnerability of astrocytic endfeet to repetitive mechanical strain, and the disruption of astrocyte-dependent glymphatic clearance together suggest that astrocytic failure may represent a foundational event upon which subsequent neuronal tauopathy is constructed. This reframing carries profound implications. If astrocytic dysfunction precedes and facilitates neuronal degeneration, then tau pathology may be better understood as a downstream manifestation of a broader collapse in neuroglial support and waste clearance. Such a model reconciles long-standing paradoxes in the field, including the spatial specificity of lesions, the delayed clinical onset following exposure, and the progressive nature of disease despite cessation of trauma. More importantly, it compels a shift in investigative focus from neurons in isolation to the integrated neurovascular and neuroglial unit as the true substrate of vulnerability in CTE.

Recognizing astrocytes as central actors in CTE pathogenesis does more than refine our understanding of this disease; instead, it expands the conceptual boundaries of traumatic neurodegeneration as a whole. It invites critical re-examination of how repetitive mechanical stress is transduced into chronic biological dysfunction and challenges the field to move beyond descriptive pathology toward mechanistic insight. Future research that interrogates astrocytic mechanotransduction, glymphatic integrity, and glial-neuronal signaling may not only illuminate the origins of CTE but also identify novel therapeutic windows such as ones that exist before irreversible neuronal loss has occurred. Ultimately, the question this body of work poses is not whether neurons matter in CTE, but whether they have been asked to carry an explanatory burden that properly belongs to the glial systems that sustain them. Reframing CTE as a disorder rooted in astrocytic failure does not diminish the role of tau; rather, it situates tau within a larger, more coherent biological narrative. In doing so, it offers a path forward, one grounded in systems biology, informed by biomechanics, and oriented toward prevention rather than post hoc recognition of disease.

## Data Availability

No datasets were generated or analysed during the current study.
